# Adverse Childhood Experiences: Relationship with Empathy and Alexithymia

**DOI:** 10.1007/s40653-023-00520-6

**Published:** 2023-02-09

**Authors:** Andreia Cerqueira, Telma Catarina Almeida

**Affiliations:** 1 Egas Moniz School of Health and Science, Instituto Universitário Egas Moniz (IUEM), LabPSI – Laboratório de Psicologia Egas Moniz, IUEM, Caparica, Portugal; 2 Egas Moniz School of Health and Science, Instituto Universitário Egas Moniz (IUEM); CiiEM – Centro de Investigação Interdisciplinar Egas Moniz, IUEM; LabPSI – Laboratório de Psicologia Egas Moniz, IUEM, Campus Universitário, Quinta da Granja, Monte de Caparica, 2829-511 Caparica, Portugal

**Keywords:** Child adversity history, Alexithymia, Empathy

## Abstract

Several studies showed that adults who have experienced childhood adversity are more likely to develop alexithymia and low empathy. Therefore, this research aims to analyze the relationship between childhood adversity and alexithymia and empathy in adulthood and verify a predictive explanatory model of alexithymia. The sample comprised 92 adults who responded to the sociodemographic questionnaire, the Childhood History Questionnaire, the Interpersonal Reactivity Index, and the Alexithymia Scale of Toronto. Childhood adversity showed a positive relationship with alexithymia and a negative relationship with empathy. Predictive validity showed that marital status, adverse childhood experiences (ACEs), and empathic concern predicted higher alexithymia scores. These results show the impact of these childhood experiences on adult life, underlining the importance of developing intervention programs in this field.

## Introduction

The context in which children or youth develop impacts their thinking and acting (Lima et al., 2019). The child’s environment is crucial for human development, influenced by negative experiences throughout life (Almeida et al., 2021; Howard, 2021; Osher et al., 2020). Studies about the history of adversity in childhood have increased to identify and understand the impact these experiences have on youth lives (Khan & Ded, 2021). According to several authors, ACEs are present in large part of the population. When ACEs, subjects are more likely to be exposed to many types of violence (e.g., Almeida et al., 2020; Nowakowski-Sims, 2019). Most of the authors report that the history of childhood adversity, such as experiences of physical abuse, emotional abuse, sexual abuse, physical neglect, emotional neglect, parental separation or divorce, mental illness or suicide in the family, household substance abuse, and incarcerated household members (Júnior, 2017), influencing social and emotional functioning (Merrick et al., 2020), health (Cardoso et al., 2018), delinquency (Pires & Almeida, 2023), and psychopathology (Clark et al., 2020). Studies carried out with young people who have experienced child adversity show that those who report child emotional abuse tend to manifest decreased emotional response, depression, inability to become independent, inability to trust others, inadequacy or emotional instability, suicide, or homicide (Finzi-Dottan & Karu, 2006).

The effects of violence can be devastating, mainly when it occurs in childhood (Gummelt, 2018; Sani & Almeida, 2020), harming a psychological level, with high levels of stress and psychological maladjustment, mainly on a social (Ferreira, 2019) and emotional level (Steinkopf et al., 2021) and even later difficulty constitution of a family, with stable interpersonal relationship (Souza & Kantorski, 2003). Some studies identify an association between the dysfunctional family environment, less emotional expressiveness, and affective inconstancy (Gusmão, 2019). Specifically, the study by Cunningham and Baker (2007) concluded that children who are victims of emotional abuse are more likely to be emotionally unstable and can maintain this pattern throughout life. This problem then affects physical and mental health, the establishment of loving relationships, and the ability to describe feelings as an adult (Holt et al., 2008). According to the multiple code theory of information, exposure to trauma during childhood can condition the child’s ability to verbalize emotions and reflect and communicate their emotional states (Bucci, 1997; Chung & Chen, 2020) showed that adolescents exposed to ACEs have difficulties with emotional processing and alexithymia.

Beyond child victimization, parental separation or divorce is also associated with alexithymia (Janiec et al., 2019). In litigious separation, a parenting style based on paternal-filial detachment is sometimes present, translated into an insecure and ambivalent connection (Lewis et al., 2000). These types of attachment are often related to difficulty identifying and dealing with emotions, which are criteria underlying the development of alexithymia (Zdankiewicz-Scigala & Scigala, 2020). Another adverse factor in childhood life linked to alexithymia is the presence of family members who consume illegal substances. This factor sometimes triggers high levels of child rejection, lack of communication, impaired emotional skills, and alexithymia, leading to conflicts and family violence (Zdankiewicz-Scigala & Scigala, 2020). The existence of a person with a mental disorder in the family may also trigger several emotional challenges (Schrank & Olschowsky, 2008), and the parents’ suicide may be related to deficits in the identification of feelings, an aspect presented by alexithymic individuals (Wheeler et al., 2005). Researchers show that individuals who have experienced the death of a parent early in life tend to develop interpersonal difficulties in adulthood (Calderon et al., 2019). Because of some of these results, Constantinidis (2017) reinforced the importance of investing in monitoring the young person and the family with an approach that facilitates communication and problem-solving during treatment. The arrest of a family member, precisely one of the parents, is also a significant aspect of developing emotional difficulties, marked by the separation between the child and the parent (Turney & Goodsell, 2018).

Those who experience adversity in childhood tend to have difficulty identifying their emotions (e.g., Thorberg et al., 2011) and difficulty recognizing emotions from the other and their point of view (e.g., Quas et al., 2017). ACEs are associated with poorer mental and physical health outcomes in adulthood (Crouch et al., 2020; Chen et al., 2021) clarify that the low quality of parental attachment, existing, for example, neglect leads to low levels of empathy and is consequently associated with adolescent depressive symptoms. However, there is no consensus on the results of studies that link these adverse events and empathy (e.g., Dargis et al., 2016). Greenberg et al. (2018) point to increased empathy in individuals with ACEs, especially in the affective components. Narvey et al. (2021) suggest that individuals with childhood adversity experience show low empathy and a high tendency to replicate deviant behaviors.

Therefore, this study aims to: (a) analyze the relationship between ACEs and alexithymia in adulthood, (b) analyze the relationship between ACEs and empathy in adulthood, (c) verify a predictive explanatory model of alexithymia.

## Method

### Participants

The sample comprised 92 Portuguese adults aged between 20 and 63 years (*M* = 40.23, *SD* = 12.14), mostly female (81.5%, *n* = 75). About 43.5% (*n* = 40) of the sample is married, 31.5% (*n* = 29) is single, 12.0% (*n* = 11) is divorced, 10.9% (*n* = 10) is in a consensual union, 1.1% (*n* = 1) is separated, and 1.1% (*n* = 1) is a widow. Regarding educational qualifications, 48.9% (*n* = 45) have higher education, 43.5% (*n* = 40) have secondary education, 4.3% (*n* = 4) have other types of qualifications, and 3.3% (*n* = 3) have basic education. The majority are employed (78.3%, *n* = 72), 8.3% are students (*n* = 8), 7.6% (*n* = 7) are unemployed, 3.3% (*n* = 3) are in another professional situation, and 2.2% (*n* = 2) are retired.

**Table 1:** Sample description

	*n*	*%*
**Gender**		
Feminine	75	81.5
Male	17	18.5
**Marital status**		
Not married	29	31.5
Non-marital partnership	10	10.9
**Separate**	1	1.1
Married	40	43.5
Divorced	11	12.0
Widower	1	1.1
**Literary abilities**		
Basic education	3	3.3
High school	40	43.9
University education	45	48.9
Other	4	4.3

*Notes. n = Number of participants; % = Percentage of participants*

### Procedures

The study design is cross-sectional, with a non-probabilistic sample collected through an online protocol disseminated by social networks. Before completing the questionnaires through the web-based survey, all participants signed an informed consent electronically. The confidentiality of results and the anonymity of subjects were ensured, and the individual would have to be an age equal to or above 18 years old and the Portuguese nationality. The protocol comprised a sociodemographic questionnaire, the questionnaire History of Adversity in Childhood (ACE: Felitti & Go, 1998; Pinto et al., 2014), the Toronto Alexithymia Scale 20 items (TAS-20: Taylor et al., 1992; Praceres et al., 2000) and the Reactivity Index Interpersonal (IRI: Davis, 1983; Limpo et al., 2010).

The study was conducted following the ethical principles outlined in the Declaration of Helsinki (World Medical Association, 2013) and the Ethical Principles of Psychologists and Code of Conduct (APA, 2017). The protocol was approved by the Institutional Review Board of the Egas Moniz School of Health and Science University, and no remuneration was granted.

### Measures

#### Sociodemographic Questionnaire

A questionnaire was used to assess sociodemographic variables: age, gender, nationality, and marital status.

#### Childhood History Questionnaire (ACE) – Short Version (Felitti & Anda, 1998; Pinto et al., 2014)

This instrument assesses the history of childhood adversity through a set of questions and statements that refer to experiences before 16 years of age. It comprises 17 items, which assess 10 factors: emotional abuse, physical abuse, sexual abuse, emotional neglect, physical neglect, parental separation or divorce, exposure to domestic violence, household substance abuse, mental illness or suicide, and incarcerated household members. ACEs are considered when the participant answers in the affirmative to at least one question of the dimension under analysis. To assess the total adversity, the scores ​​in each dimension are added. The instrument has a good internal consistency in the validation (*α* = 0.88) and this study (*α* = 0.80).

#### Toronto Alexithymia Scale (TAS-20; Taylor et al.,^1992^; Praceres et al., 2000)

This scale consists of 20 items for evaluating characteristics of the three alexithymia: difficulty identifying feelings and distinguishing them from the bodily sensations of emotion that assess the difficulty in recognizing feelings and distinguishing them from bodily sensations resulting from emotional activation (e.g., “I have feelings that I can’t quite identify”; difficulty describing feelings measure the difficulty in expressing feelings (e.g., “I have trouble finding the right words to describe my feelings”, and externally orientated thinking to evaluate cognitive style, with external guidance (e.g., I prefer to talk to people about their daily activities than about their feelings”). In the TAS-20 validation study (Praceres et al., 2000), both the total scale (*α* = 0.79) and the dimensions showed levels of adequate internal consistency. The Cronbach’s alpha in our study was good (*α* = 0.82).

#### Reactivity Index Interpersonal (IRI; Davis, 1983; Limpo et al., 2010)

This instrument assesses empathy through a set of statements that describe feelings and thoughts. It comprises 24 statements that evaluate four factors: perspective-taking, which reflects the tendency to adopt the other person’s point of view (e.g., Before criticizing someone, I try to imagine how I would feel if I were in their place.”); empathic concern, refers to the ability to experience feelings of compassion and concern for others (e.g., “When I see someone being taken advantage of, I feel like protecting them.”); personal distress assesses feelings of anxiety, apprehension, and discomfort in interpersonal contexts of tension (e.g., “In emergency situations, I feel uncomfortable and apprehensive/apprehensive.”), and fantasy refers to the individual’s propensity to put himself in fictional situations (e.g., “I easily get involved in the feelings of the characters in a novel.”). The instrument has a good internal consistency reliability coefficient in the validation study, and the dimensions showed adequate internal consistency in the original study (*α* = 0.96) and our study (*α* = 0.70).

### Statistical Analysis

To perform the data analysis, the statistical software IBM statistical version of the SPSS was used (v.27.0). First, descriptive statistics for the sociodemographic variables, the Ace, the TAS-20, and the IRI were calculated. Next, Pearson’s correlations and Cramer’s V analyzes were performed on the dichotomous variables under study that is found in the ACE instrument. The four variables were evaluated only by one item of dichotomous response (parental separation or divorce, household substance abuse, and mental illness or suicide in the family and incarcerated household members). According to Marôco (2021), the correlations in dichotomous items should be analyzed through Cramer’s V commands. The remaining variables were analyzed using Pearson’s correlations. Multiple linear regressions were also performed to analyze the predictive validity of alexithymia.

## Results

### Descriptive Analysis

Descriptive analysis showed that 64.1% (*n* = 59) of participants witnessed episodes of violence in childhood. Concerning the ACE total score (see Table 2), 67.4% of the participants identified a history of adverse experiences in childhood. The most prevalent experiences reported were emotional abuse (28.3%), emotional neglect (25%), physical abuse (23.9%), and mental illness or suicide in the family (23.9%). Concerning Tas-20, all the participants recognized alexithymia. Externally oriented thinking was the dimension with the highest expression (100%). According to the IRI results, all the participants identified empathic ability.

**Table 2:** Descriptive Statistics of the ACE, TAS-20, IRI and dimensions

Scale and Dimentions	*n*	%	*M*	*SD*
ACE	62	67.4	2.46	3.12
Emotional abuse	26	28.3	0.40	0.70
Physical abuse	22	23.9	0.33	0.63
Sexual abuse	13	14.1	0.22	0.58
Emotional neglect	23	25	0.38	0.71
Physical neglect	2	2.2	0.04	0.30
Parental separation or divorce	11	12.0	0.12	0.37
Exposure to domestic violence	10	10.9	0.51	0.58
Household substance abuse	17	18.5	0.18	0.39
Mental illness or suicide in the family	22	23.9	0.24	0.43
Incarcerated household members	3	3.3	0.03	0.18
Tas-20	92	100	53.21	12.21
Difficulty identifying feelings	85	78.3	13.90	6.67
Difficulty describing feelings	91	98.9	13.02	3.87
Externally oriented thinking	92	100	26.28	5.27
IRI	92	100	57.97	9.79
Perspective taking	92	100	16.87	3.41
Empathic concern	92	100	16.85	2.88
Personal distress	92	100	12.24	3.49
Fantasy	92	100	12.01	3.85

*Notes. M = Mean; SD = Standard deviation*

### Correlational Analysis

The results concerning the correlations between the ACE scale and TAS-20 scale show the existence of a statistically significant positive correlation between emotional neglect in childhood and the difficulty in identifying feelings and distinguishing them from the bodily sensations of emotion (*r* = .27, *p* = .01), the difficulty in describing feelings to others (*r* = .36, *p* < .001), and externally orientated thinking (*r* = .22, *p* = .03).

The correlational results between the ACE scale and the IRI point to statistically significant negative correlations between emotional abuse and empathic concern (*r* = − .30, *p* < .001), physical abuse and taking perspective (*r* =-. 28, *p* < .001), emotional neglect and taking perspective in adulthood (*r* = − .23, *p* = .03). We can also verify a relationship between the incarcerated household members and the taking perspective (*v* = 0.72, *p* < .001) and the incarcerated household members and the personal distress (*v* = 0.59, *p* = .012).

**Table 3:** Correlation between ACE, TAS-20 e IRI

	1.	2.	3.	4.	5.	6.	7.	8.	9.	10.	11.	12.	13.
**ACE** **1.** Emotional abuse	-	0.80	− 0.03	0.43	0.13	0.11	0.01	0.17	0.00	− 0.09	− 0.30*	− 0.09	− 0.02
**2.** Physical abuse		-	0.08	0.41	0.40	0.07	0.09	0.14	0.07	− 0.28*	− 0.20	− 0.16	− 0.03
**3.** Sexual abuse			-	0.07	0.33	0.05	0.11	0.13	0.07	− 0.17	0.01	0.10	0.05
**4.** Emotional neglect				-	0.34	0.16	0.27*	0.36*	0.22*	− 0.23*	− 0.11	− 0.02	0.02
**5.** Physical neglect					-	0.24	− 0.03	− 0.07	0.10	− 0.13	0.06	− 0.03	− 0.04
**6.** Exposure to domestic violence						-	− 0.15	− 0.10	− 0.30	− 0.09	0.20	− 0.12	− 0.15
**TAS-20** **7.** Difficulty identying feelings							-	0.82	0.16	− 0.40	0.16	0.30	0.21
**8.** Difficulty describing geelings								-	0.16	0.34	0.25	0.23	0.08
**9.** Externally oriented thinking									-	0.34	0.20	0.23	− 0.00
**IRI** **10.** Perspective taking										-	0.40	0.34	0.34
**11.** Empathic concern											-	0.32	0.38
**12.** Personal distress												-	0.34
**13.** Fantasy													-

*Notes*. *p < .001

### Regression Analysis

The explanatory model of alexithymia using a Multiple Linear Regression showed that the model is significant. Durbin-Watson was 1.76, and VIF was < 3. Age, emotional abuse, household substance abuse, perspective-taking, and fantasy are not significant, and for this reason, we performed a new model (Table 3) only with significant paths. The model is significant (*F*(10,37) = 15.50, *p* ≤ .001) and explains 76% of the variance of alexithymia. Marital status (β = -0.49, *p* ≤ .001), physical abuse (β = 0.37, *p* = .001), sexual abuse (β = 0.36, *p* ≤ .001), emotional neglect (β = 0.39, *p* ≤ .001), physical neglect (β = -0.32, *p* = .001), parental separation or divorce (β = -0.28, *p* = .005), exposure to domestic violence (β = 0.19, *p* = .028), mental illness or suicide in family (β = -0.21, *p* = .013), incarcerated household members (β =-0.24, *p* = .003), and empathic concern (β = 0.55, *p* ≤ .001) are significant predictors of alexithymia.

**Table 4:** Multiple Linear Regression with Alexithymia

Variable	β	t	*p*	Adj R^2^	∆R^2^	F	
				0.81	0.76	* F*(10,37) = 15.50, *p* ≤ .001	
Marital status	-0.49	-5.39	≤ 0.001				
Physical abuse	0.37	3.48	0.001				
Sexual abuse	0.36	4.30	≤ 0.001				
Emotional neglect	0.39	4.49	≤ 0.001				
Physical neglect	-0.32	-0.37	0.001				
Parental separation/divorce	-0.28	3.02	0.005				
Exposure to domestic violence	0.19	2.29	0.028				
Mental illness or suicide in the family	-0.21	-2.61	0.013				
Incarcerated household members	-0.24	-3.18	0.003				
Empathic concern	0.55	6.71	≤ 0.001				

## Discussion

The results showed that most of the sample had witnessed violent events; almost half of the sample had been victims of violence. These results are consistent with the figures provided by the Portuguese Association for Victim Support in 2019, which identified 11,676 crime victims. Besides, the Annual Homeland Security Report for 2019 showed high values ​​of general and violent crime, referencing 350,012 actions and a high percentage of victimization.

Concerning the history of childhood adversities, as described by the sample, the most common factor identified in our sample is emotional abuse. This result follows the study of Merrick et al. (2018), which identified emotional abuse in the family as the most frequently reported factor in the history of childhood adversity. This result reflects the Portuguese reality since, through the statistics revealed by APAV in 2020, emotional and physical abuse are the most reported victimizations by victims.

Regarding alexithymia, the most described factor by the sample in this study was thought directed outwards, supporting the study of Praceres et al. (2000), the authors responsible for validating the Portuguese version of the alexithymia instrument. This factor is related to recognizing facial emotions, a concrete operation with a cognitive dimension (Pinto et al., 2018). Perspective-taking was the factor that showed the highest values ​​and corresponded to a cognitive dimension (Cruz, 2018). This result does not support the study of validation of the instrument in the Portuguese population. Limpo et al. (2010) stated that empathic concern is the most prevalent factor. This difference in results may be explained by the sample characteristics of the two studies, which in the authors’ research is homogeneous about gender and the present study presents a considerably higher number of women. The average age of the samples is also different since our sample comprises the oldest people. According to Engelberg and Limbach-Reich (2015), women tend to have the highest scores of perspectives, and according to Rieffe et al. (2010), older people tend to show higher scores of empathy with greater ease in maintaining interpersonal relationships.

The data from our research highlighted the relationship between a history of childhood adversity and a lack of empathy concerning emotional abuse, physical abuse, and emotional neglect. The study by Del Prette and Del Prette (2003) also shows this relationship, showing low empathy scores in individuals who grew up in negligent and abusive environments. Komatsu and Bazon (2018) also highlighted ACEs as risk factors for developing low empathy. The youth witnesses will influence their behavior patterns in adulthood. Adverse experiences also influence brain evolution (Oliveira et al., 2010) and the network responsible for empathy (Abraham et al., 2018). In our study, the arrest of a family member was also related to low empathic competence, a result also verified by Wilbur et al. (2007). These results can be explained by the separation between the child and the incarcerated, having even more impact if the latter is the father or the mother. According to other studies, empathy develops through a secure attachment. Thus, with parental imprisonment, there is an abrupt separation and rupture of the child’s sense of security, influencing their empathy development (Thomsom et al., 2017).

In our study, emotional neglect was linked to alexithymia, such as difficulty identifying and describing feelings and thinking oriented toward the outside. These results are corroborated by Yoshida (2005) since the author mentioned that childhood neglect affects the development of alexithymia in adulthood. In this situation, as in the incarcerated household members, the support and attachment network are scarce, and the child’s social development tends to be the poorest (Herrero-Roldán et al., 2019).

Considering the history of adversity in childhood and committing aggressive behaviors in adulthood, it is possible to verify in our and other studies (e.g., King, 2021) that individuals who experienced victimization in childhood tend to show a higher probability of presenting aggressive behavior in adulthood. Those children tend to have more problems during school, delinquent behaviors, social maladjustments, and even influencing personality traits (Farrell et al., 2017), which can explain our results.

The previous results also show that emotional neglect explains variances in the difficulty of describing feelings, taking perspective, and explaining the increased probability of threatening third parties. These results are supported by the study of Reis et al. (2018), which explained that the neglect experienced by children in the family may justify the difficulties in the relationship with others and that there is a greater probability of the child developing aggressive behaviors. The same applies to emotional abuse, which varies with empathic concern and threatening behaviors. Reis et al. (2018) also showed that hostile, adverse, abusive, and negligent environments could lead to aggressive behaviors. Physical abuse is linked to a variance in perspective and the threat to others, which is also explained by Sales et al. (2020), clarifying that physical abuse can lead to violent behaviors and more significant difficulties in perceiving rules of socialization and conduct. The incarcerated household members lead to its absence, and this factor explains variances in perspective, which according to Altintase and Bilici (2018), has implications for difficulties relating to others. Finally, threatening behaviors tend to lead to variances of difficulty in identifying feelings. This finding is corroborated by Conde and Teixeira (2017), who showed the link between delinquent behaviors and difficulty in relating to other people because of personal characteristics and weak emotional control.

As described above, several factors predict alexithymia. As verified in our results through linear analysis, multiple instabilities in the family environment, such as divorce/parental separation, domestic violence, neglect, and physical abuse, are some of these predictors, which agrees with studies carried out by Villanueva and Benavides (2019). Sexual abuse is also cited as an explanatory variable of alexithymia, identified by Carneiro and Yoshida (2009). Several studies point to these variables as environmental variables, as predictors of problems in the circuit of emotions, specifically about recognizing feelings and respective responses (Teixeira et al., 2019). The indicial studies identified that most subjects with alexithymia had experienced traumatic events throughout their lives, which are predictors of alexithymia (Zeitlan & McNally, 1993).

This empirical work can answer some essential ways to intervene with victims of adversities in childhood, reducing possible consequences both at a psychological and behavioral level.

### Limitations

Our research has some limitations that should be considered in future studies. The first limitation of this study is related to the sample size. A sample with smaller dimensions, composed mainly of women and not representative of our population, makes it impossible to generalize the results. Thus, it is necessary to increase the sample in future studies, making it more homogeneous regarding gender. Filling in the online protocol can also be a limitation since it is impossible to control the environment in which the subject fills in the instruments and how often he does it. Also, it is necessary to consider social desirability when completing the protocol since this is a sensitive topic.

To the limitations described above, it is possible to verify that the study got some results that do not corroborate the literature, which reveals a need to assess whether other variables may influence the results, such as the respondent’s culture, the language used, the adequacy of the instruments used, and the social desirability associated with completing self-assessment instruments. In future studies, it would also be necessary to compare individuals with and without a history of adversity in childhood.

Despite the limitations mentioned above, this study verified that alexithymia and empathy are associated with a history of childhood adversities. It is also possible to assess those suffering violent events in childhood, either a relationship with alexithymia.

### Implications for Practice

In the present study, relevant results for Psychology are found since we identified a relationship between the studied variables, allowing us to expand the knowledge about human behavior and the factors that can influence it at the emotional and behavioral levels. Some recent studies identify the negative impact of childhood experiences across lifelong (e.g., Almeida et al., 2023a, b; Pires & Almeida, 2023), and other ones identify some protective factors to develop a healthy life in adulthood (e.g., Almeida et al., 2023b; Letourneau et al., 2020). Discovering potential risk and protective factors is essential to create intervention programs that can be implemented, for example, in the National Justice System, with victims and offenders.

It is possible to identify that many of the sample experienced abuse and neglect in childhood. Following this, we can suggest an intervention. According to some studies with abused and neglected children, motivational interviews, cognitive behavioral therapy, family therapy, and psychodynamic psychotherapy can be efficient in children under 18 years old. Therapy performed at school to intervene with abused and neglected children must adopt an individualized care plan. These psychological interventions aim to improve their mental resilience (e.g., Lorenc et al., 2020).

Another of the most referenced situations by the participants in our sample is mental illness or suicide in the family. The Portuguese population is one of the Europe countries showing the highest levels of some disorders, with anxiety being the most prominent (Chyczij et al., 2020). Children of parents with mental disorders are also more likely to develop mental problems (Nascimento, 2001) and emotional regulation difficulties (Martins et al., 2021). Psychotherapy through psychoeducation groups with children and young people can effectively reduce anxiety symptoms and inappropriate and violent behavior (Lorenc et al., 2020).

Furthermore, parents with mental health problems, such as depression and living in a violent environment, show greater difficulty parenting young children (Anis et al., 2022). However, their capacity to understand their children’s feelings and to create a healthy attachment with them can buffer the negative impact of some life stressors (Letourneau et al., 2020). Furthermore, positive parenting positively impacts children’s emotional skills (Martins et al., 2022), which may create resilience in the face of the adversities experienced and minimize the possibility of developing alexithymia. The development of positive parenting and positive parent-child relationship programs can be an added value to reducing mental health problems that come from adverse childhood experiences and behavioral problems in children, enhancing their well-being and health. In this way, developing projects to provide the population with the capability to develop parent-child attachment (Anis et al., 2022) and positive parenting predict lower child abuse risk over time (Gonzalez & Rodriguez, 2023).

Another way to decrease childhood victimization may be to train primary and secondary education teachers and health technicians to identify characteristics referring to these types of victims that would allow the referral of these young people for intervention, decreasing the negative consequences of adverse life experiences. Awareness actions in schools for youth can also be necessary. Those actions could approach subjects related to child victimization, feelings identification, and mental health problems in young people, providing them solutions in the community (e.g., victim support, psychological intervention). Those actions intend to raise awareness about seeking help and combating violence.

This knowledge can also be applied in advertising campaigns so that the general population knows the consequences of child victimization, minimizing the damage from this problem. Furthermore, this study opens lines of research that are still not very explored.

## References

[CR1] Abraham E, Raz G, Zagoory-Sharon O, Feldman R (2018). Empathy networks in the parental brain and their long-term effects on children’s stress reactivity and behavior adaptation. Neuropsychologia.

[CR2] Almeida TC, Fernandes RM, Cunha O (2023). Brief measure of affective lability among Portuguese Community and Justice samples: Psychometrics and Measurement Invariance. Crime & Delinquency.

[CR3] Almeida, T. C., Fernandes, R. M., & Cunha, O. (2023b). The role of positive childhood experiences in the link between childhood maltreatment and affective lability in a sample of incarcerated men and women. *Child Abuse & Neglect*, 105969. 10.1016/j.chiabu.2022.105969. 10.1016/j.chiabu.2022.105969 36436298

[CR4] Almeida, T. C., Guarda, R., & Cunha, O. (2021). Positive childhood experiences and adverse experiences: psychometric properties of the Benevolent Childhood Experiences Scale (BCEs) among the portuguese population. *Child Abuse & Neglect*, 120. 10.1016/j.chiabu.2021.105179. 10.1016/j.chiabu.2021.105179 34198123

[CR5] Almeida, T. C., Ramos, C., Brito, J., & Cardoso, J. (2020). The Juvenile victimization questionnaire: psychometric properties and polyvictimization among portuguese youth. *Children and Youth Services Review*, *113*, 10.1016/j.childyouth.2020.105001.

[CR6] Altintas M, Bilici M (2018). Evaluation of childhood trauma with respect to criminal behavior, dissociative experiences, adverse family experiences and psychiatric backgrounds among prison inmates. Comprehensive psychiatry.

[CR7] American Psychological Association [APA]. (2017). *American Psychological Association’s (APA) ethical principles of psychologists and code of conduct*. American Psychology Association.

[CR90] Anis, L., Ross, K., Ntanda, H., Hart, M., & Letourneau, N. (2022). Effect of Attachment and Child Health (ATTACHTM) Parenting Program on Parent-Infant Attachment, Parental Reflective Function, and Parental Depression. *International Journal of Environmental Research and Public Health, 19*(14), 8425. 10.3390/ijerph19148425 10.3390/ijerph19148425 PMC9324434 35886276

[CR8] Associação Portuguesa de Apoio à Vítima – APAV (2019). Estatísticas APAV - Relatório Anual 2018 [APAV Statistics - Annual Report 2018]. Lisboa: Associação Portuguesa de Apoio à Vítima. https://apav.pt/apav_v3/images/pdf/Estatisticas_APAV-Relatorio_Anual_2019.pdf

[CR9] Associação Portuguesa de Apoio à Vítima – APAV (2020). Estatísticas APAV - Relatório Anual 2018 [APAV Statistics - Annual Report 2018]. Lisboa: Associação Portuguesa de Apoio à Vítima. https://apav.pt/apav_v3/images/pdf/Estatisticas_APAV_Relatorio_Anual_2020.pdf

[CR10] Bucci W (1997). Symptoms and symbols: a multiple code theory of somatization. Psychoanalytic Inquiry.

[CR11] Calderon S, Samstag LW, Papouchis N, Saunders BA (2019). The Effects of early parental death and grief on interpersonal functioning and alexithymia in adults. Psychopathology.

[CR12] Cardoso J, Almeida TC, Ramos C, Sousa S (2018). Relationship between childhood trauma and sleep disturbances: the role of perceived stress as a mediator. Journal of Aggression Maltreatment & Trauma.

[CR13] Carneiro BV, Yoshida EMP (2009). Alexitimia: Uma revisão do conceito. Psicologia: Teoria e Pesquisa.

[CR14] Chen, H., Meza, J. I., Yan, Y., Wu, Q., & Lin, X. (2021). Parental attachment and depression in adolescents: moderation mediation model of empathy and gender. *Current Psychology*, 1–12. 10.1007/s12144-021-01698-4.

[CR16] Chung MC, Chen ZS (2020). Gender differences in child abuse, emotional processing difficulties, alexithymia, psychological symptoms and behavioural problems among chinese adolescents. Psychiatric quarterly.

[CR17] Chyczij FF, Ramos C, Santos AL, Jesus L, Alexandre JP (2020). Prevalência da depressão, ansiedade e stress numa unidade de saúde familiar no norte de Portugal [Prevalence of depression, anxiety and stress in a family health unit in northern Portugal]. Revista de Enfermagem Referência.

[CR18] Clark, R., Menna, R., McAndrew, A. J., & Johnson, E. M. (2020). Language, Aggression, and self-regulation in Young Children. *Journal of Emotional and Behavioral Disorders*, 1063426620937691. 10.1177/1063426620937691.

[CR19] Conde, R., & Teixeira, S. (2017). Histórias de vida de jovens delinquentes: O contributo da investigação qualitativa para a compreensão da Delinquência Juvenil [Life stories of young delinquents: The contribution of qualitative research to the understanding of juvenile delinquency]. *CIAIQ 2017*, *3*.

[CR20] Constantinidis TC (2017). Profissionais de saúde mental e familiares de pessoas com sofrimento psíquico: Encontro ou desencontro [Mental health professionals and family members of people with psychic suffering: meeting or missing each other?]?. Psicologia USP.

[CR21] Crouch, E., Jones, J., Strompolis, M., & Merrick, M. (2020). Examining the Association between ACEs, Childhood Poverty and Neglect, and physical and Mental Health: data from two state samples. *Children and Youth Services Review*, 105155. 10.1016/j.childyouth.2020.105155.

[CR22] Cruz, S. D. D. (2018). *Influência dos Níveis de Empatia e dos Sintomas Psicopatológicos na Capacidade de Reconhecimento Facial*. [Influence of Empathy Levels and Psychopathological Symptoms on Facial Recognition Capacity] Doctoral dissertation. Universidade da Beira Interior, Covilh&#227

[CR23] Cunningham AJ, Baker LL (2007). Little eyes, little ears: how violence against a mother shapes child as they grow.

[CR24] Dargis M, Newman J, Koenigs M (2016). Clarifying the link between childhood abuse history and psychopathic traits in adult criminal offenders. Personality Disorders: Theory Research and Treatment.

[CR25] Davis MH (1983). Measuring individual differences in empathy: evidence for a multidimensional approach. Journal of Personality and Social Psychology.

[CR26] Del Prette, A., Prette, D., Z. A. P., & Villa, M. B. (2003). Habilidades sociais, desenvolvimento e aprendizagem: Questões conceituais, avaliação e intervenção [Social skills, development, and learning: Conceptual issues, assessment, and intervention].Campinas: Alínea,259–291.

[CR27] Engelberg E, Limbach-Reich A (2015). The role of empathy in case management: a pilot study. Social Work Education.

[CR28] Farrell AD, Thompson EL, Mehari KR (2017). Dimensions of peer influences and their relationship to adolescents’ aggression, other problem behaviors and prosocial behavior. Journal of Youth and Adolescence.

[CR29] Felitti V, Anda R, Nordenberg D, Williamson D, Spitz A, Edwards V, Marks J (1998). Childhood trauma tied to adult illness. American Journal of Preventative Medicine.

[CR30] Ferreira SO (2019). Emotional activation in human beings: procedures for experimental stress induction. Psicologia USP.

[CR31] Finzi-Dottan R, Karu T (2006). From emotional abuse in childhood to psychopathology in adulthood: a path mediated by immature defense mechanisms and self-esteem. The Journal of Nervous and Mental Disease.

[CR33] Gonzalez S, Rodriguez CM (2023). Psychosocial Resources Predicting maternal and paternal positive parenting and lower child abuse risk. Prevention Science: The Official Journal of the Society for Prevention Research.

[CR34] Greenberg DM, Baron-Cohen S, Rosenberg N, Fonagy P, Rentfrow PJ (2018). Elevated empathy in adults following childhood trauma. PLoS one.

[CR35] Gummelt G (2018). “Violence is everywhere!” An analysis of children’s narratives on violence. Child & Youth Services.

[CR36] Gusmão, A. C. C. D. (2019). *Disfuncionalidade familiar durante a infância e a adolescência e o desenvolvimento de traços antissociais [Family dysfunctionality during childhood and adolescence and the development of antisocial trait]* Doctoral dissertation. Instituto Universitário Ciências Psicoloógicas, Sociais e da Vida, Lisboa.

[CR38] Herrero-Roldán S, Byrne S, Rodrigo MJ, Hernández-Cabrera JA, León I (2019). Sesgos en la evaluación del llanto infantil en la negligencia materna: el papel de la alexitimia [Biases in the evaluation of infant crying in maternal neglect: the role of alexithymia]. Revista de estudios e investigación en psicología y educación.

[CR39] Holt S, Buckley H, Whelan S (2008). The impact of exposure to domestic violence on children and young people: a review of the literature. Child Abuse & Neglect.

[CR40] Howard, S. (2021). A causal model of children’s vicarious traumatization. *Journal of Child & Adolescent Trauma*, 1–12. 10.1007/s40653-020-00331-z. 10.1007/s40653-020-00331-z PMC7779647 33425092

[CR41] Janiec M, Toś M, Bratek A, Rybak E, Drzyzga K, Kucia K (2019). Family and demographic factors related to alexithymia in polish students. Archives of Psychiatry and Psychotherapy.

[CR42] Júnior, G. L. S. (2017). *A influência da afetividade sobre a associação entre adversidades na infância e patologia da personalidade na vida adulta [The influence of affectivity on the association between adversity in childhood and personality pathology in adulthood]* Master’s Thesis. Faculdade de Medicina da Universidade de São Paulo, São Paulo. 10.11606/T.5.2018.tde29012018-131957

[CR44] Khan A, Deb A (2021). Family as a source of risk and resilience among adults with a history of childhood adversity. Children and Youth Services Review.

[CR45] King AR (2021). The ACE questionnaire and lifetime physical aggression. Journal of Aggression Maltreatment & Trauma.

[CR46] Komatsu, A. V., & Bazon, M. R. (2018). Fatores de risco e de proteção para emitir delitos violentos: revisão sistemã tica da literatura [Risk and protective factors for issuing violent crimes: systematic review of the literature]. *Perspectivas em Psicologia*, *22*(1), 10.14393/PPv22n1a2018-13.

[CR47] Letourneau N, Anis L, Ntanda H, Novick J, Steele M, Steele H, Hart M (2020). Attachment & Child Health (ATTACH) pilot trials: Effect of parental reflective function intervention for families affected by toxic stress. Infant Mental Health Journal.

[CR48] Lewis M, Feiring C, Rosenthal S (2000). Attachment over time. Child Development.

[CR49] Lima, J. F., Gomes, A. T., & de Souza, R. M. (2019). The impacts of the profile of the youth and adult education student on the continuing education of teachers in the municipal network of Manaus/Amazonas]. *Revista Saberes & Práticas*, *1*, 23–37. Os impactos do perfil do aluno da educação de jovens e adultos sobre a formação continuada de professores da rede municipal de Manaus/Amazonas [.

[CR50] Limpo T, Alves RA, Castro SL (2010). Medir a empatia: Adaptação portuguesa do Índice de Reactividade Interpessoal [Measuring empathy: portuguese adaptation of the interpersonal reactivity index]. Laboratório de Psicologia.

[CR52] Lorenc T, Lester S, Sutcliffe K, Stansfield C, Thomas J (2020). Interventions to support people exposed to adverse childhood experiences: systematic review of systematic reviews. BMC public health.

[CR54] Martins, S., Augusto, C., Silva, M., Duarte, A., Martins, M. D. F. D. S. V., & Rosário, R. (2022). Parentalidade positiva e a sua relação com o desenvolvimento socioemocional em crianças [Positive parenting and its relationship with socio-emotional development in children]. *Revista de Estudios e Investigación en Psicología y Educación*, *9*, 118–131. 10.17979/reipe.2022.9.0.8908

[CR55] Martins CR, Neiva ACL, Bahia AF, Oliveira CX, Cardoso MIS, Abreu JN (2021). Parents’ mental health and children’s emotional regulation during the COVID-19 pandemic. Psicologia: Teoria e Prática.

[CR92] Marôco, J. (2021). *Análise Estatística com o SPSS Statistics (8? ed.) [Statistical Analysis with SPSS Statistics (8th ed.)]*. ReportNumber, Lda.

[CR56] Merrick MT, Ford DC, Ports KA, Guinn AS (2018). Prevalence of adverse childhood experiences from the 2011–2014 behavioral risk factor surveillance system in 23 states. JAMA pediatrics.

[CR91] Merrick, J. S., Narayan, A. J., Atzl, V. M., Harris, W. W., & Liberman, A. F. (2020). Type versus timing of adverse and benevolent childhood experiences for pregnant women’s psychological and reproductive health. *Children and Youth Services Review, 114*, 1–11. 10.1016/j.childyouth.2020.105056

[CR57] Narvey C, Yang J, Wolff KT, Baglivio M, Piquero AR (2021). The interrelationship between empathy and adverse childhood experiences and their impact on juvenile recidivism. Youth Violence and Juvenile Justice.

[CR58] Nascimento CRR (2001). Relações entre a resposta de ansiedade de pais e mães e a reposta de ansiedade de seus filhos [Relationships between the anxiety response of fathers and mothers and the anxiety response of their children]. Estudos de Psicologia (Campinas).

[CR59] Nowakowski-Sims E (2019). An exploratory study of childhood adversity and delinquency among youth in the context of child-to-parent and sibling-to-sibling violence. Journal of Family Social Work.

[CR61] Oliveira PAD, Scivoletto S, Cunha PJ (2010). Estudos neuropsicológicos e de neuroimagem associados ao estresse emocional na infância e adolescência [Neuropsychological and neuroimaging studies associated with emotional stress during childhood and adolescence]. Archives of Clinical Psychiatry (São Paulo).

[CR62] Osher D, Cantor P, Berg J, Steyer L, Rose T (2020). Drivers of human development: how relationships and context shape learning and development1. Applied Developmental Science.

[CR63] Pinto, L. C., Souto, T., Ribeiro, Ó., Alves, H., & Dias, R. (2018). *Alexitimia e envelhecimento de consumidores de substâncias psicoativas. [Alexithymia and aging of consumers of psychoactive substances]*. Direitos sociais e exclusão.

[CR64] Pinto R, Correia L, Maia Â (2014). Assessing the reliability of retrospective reports of adverse childhood experiences among adolescents with documented childhood maltreatment. Journal of Family Violence.

[CR65] Praceres N, Parker DA, Taylor GJ (2000). Adaptação Portuguesa da Escala de Alexitimia de Toronto de 20 itens (TAS-20) [Portuguese adaptation of the 20-Item Toronto Alexithymia Scale (TAS-20)]. Revista Iberoamericana de Diagnóstico y Evaluación Psicológica.

[CR66] Pires AR, Almeida TC (2023). Risk factors of poly-victimization and the impact on Delinquency in Youth: a systematic review. Crime & Delinquency.

[CR67] Quas JA, Dickerson KL, Matthew R, Harron C, Quas CM (2017). Adversity, emotion recognition, and empathic concern in high-risk youth. PloS One.

[CR68] Reis, D. M., Prata, L. C. G., & Parra, C. R. (2018). O impacto da violência intrafamiliar no desenvolvimento psíquico infantil [The impact of intrafamily violence on children’s psychic development]. *Psicologia: O portal dos psicólogos*. https://creativecommons.org/licenses/by-nc-nd/4.0/

[CR69] Rieffe C, Ketelaar L, Wiefferink CH (2010). Assessing empathy in young children: construction and validation of an empathy questionnaire (EmQue). Personality and Individual Differences.

[CR70] Sales SS, Knapik J, Cruz RM (2020).

[CR71] Sani, A., & Almeida, T. C. (2020). Assessing young children’s perception of interparental conflicts: The Portuguese version of the CPIC-Y and its utility. In A. M. Columbus, *Advances in Psychology Research (Vol. 142)*: Nova Science

[CR72] Schrank G, Olschowsky A (2008). Centers of psycho-social attention and the strategies for family insertion. Revista da Escola de Enfermagem da USP.

[CR74] Souza, G. L., & Kantorski, L. P. (2003). Maus tratos na infância [Childhood abuse]. *Família, Saúde e Desenvolvimento, 5*(3), 213–222. 10.5380/fsd.v5i3.8084

[CR75] SSI (2019). Relatório Anual de Segurança Interna 2018 [2018 Annual Homeland Security Report]. Lisboa: SSI. https://www.portugal.gov.pt/download-ficheiros/ficheiro.aspx?v=%3d%3dBQAAAB%2bLCAAAAAAABAAzNDA0sAAAQJ%2bleAUAAAA%3d

[CR76] Steinkopf, H., Nordanger, D., Stige, B., & Milde, A. M. (2021). Experiences of becoming emotionally dysregulated. A qualitative study of staff in Youth Residential Care. *Child & Youth Services*, 1–19. 10.1080/0145935X.2021.1918541.

[CR77] Taylor GJ, Bagby M, Parker JD (1992). The revised Toronto Alexithymia Scale: some reliability, validity, and normative data. Psychotherapy and Psychosomatics.

[CR78] Teixeira S, Barbosa JP, Lourenço L, Gonçalves D, Guardiano M (2019). Avaliação da Alexitimia em Crianças Portuguesas com Perturbação de Hiperatividade/Défice de Atenção [Assessment of Alexithymia in Portuguese Children with attention deficit hyperactivity disorder]. Gazeta Médica.

[CR80] Thorberg FA, Young RM, Sullivan KA, Lyvers M, Connor JP, Feeney GF (2011). Alexithymia, craving and attachment in a heavy drinking population. Addictive Behaviors.

[CR81] Turney K, Goodsell R (2018). Parental incarceration and children’s wellbeing. The Future of Children.

[CR82] Villanueva, C., & Benavides, V. (2019). Funcionamiento familiar y alexitimia en estudiantes con sobrepeso u obesidad de una Universidad Privada de Arequipa. Master’s Thesis. University of Arequipa

[CR83] Wheeler K, Greiner P, Boulton M (2005). Exploring alexithymia, depression, and binge eating in self-reported eating disorders in women. Perspectives in Psychiatric Care.

[CR84] Wilbur MB, Marani JE, Appugliese D, Woods R, Siegel JA, Cabral HJ, Frank DA (2007). Socioemotional effects of fathers’ incarceration on lowincome, urban, school-aged children. Pediatrics.

[CR85] World Medical Association (2013). World medical association declaration of helsinki: ethical principles for medical research involving human subjects. Journal Of The American Medical Association.

[CR86] Yoshida, E. M. P. (2005). Stress e alexitimia [Stress and alexithymia]. In *Anais do II Congresso Brasileiro de Stress*.

[CR87] Zdankiewicz-Ścigała E, Ścigała DK (2020). Attachment style, early childhood trauma, alexithymia, and dissociation among persons addicted to alcohol: structural equation model of dependencies. Frontiers in psychology.

[CR88] Zdankiewicz-Ścigała E, Ścigała DK (2020). Attachment style, early childhood trauma, alexithymia, and dissociation among persons addicted to alcohol: structural equation model of dependencies. Frontiers in psychology.

[CR89] Zeitlan SB, McNally RJ (1993). Alexithymia and anxiety sensitivity in panic disorder and obsessive- compulsive disorder. American Journal of Psychiatry.

